# Case of Abscess of the Liver, in Which the Matter Was Evacuated by Incision

**Published:** 1825-01

**Authors:** C. F. Vandeburgh

**Affiliations:** Surgeon, Royal Navy.


ART. I.—*Case of Abscess of the Liver, in which the Matter was evacuated by Incision.* By C. F. VANDEBURGH, M.D. Surgeon, Royal Navy.

JOHN TALBERT, an American seaman, ætatis thirty-six, of a robust make, but much emaciated, (had been following the advice of an empiric for more than three weeks, under the promise of a cure being effected in a few days,) was placed under my care on the 6th day of August, 1821: complaining of severe pain in the left hypochondrium, extending to the scapula of the right side; a heavy sensation, with difficulty and increase of pain at respiration; inability to lie on either side; short cough, general languor, and debility; a yellow suffusion over the body and eyes; constant inclination to vomit; speech low; bowels costive, urine tinged yellow; pulse 110, and contracted; tongue parched, great thirst, and alternate sensation of heat and cold. On examining the hypochondriac region, it appeared tense and hard: the slightest pressure augmented the pain, also stooping or extending the body forward, flying up to the clavicle. The patient observed, the pain had been, more cr less, for eighteen months, when he received a blow under the cartilago insiformis, at New Orleans: has ever since had a bitter taste in his mouth, accompanied with loss of appetite; frequently took a vomit while at sea, being generally relieved after emitting. He has been under the care of several professional men, who considered his complaint indigestion, but never received permanent benefit. For many years he has been employed in the East Indies, where, agreeable to his own phrase, he was "once liver-grown, but was perfectly restored." —Venesectio ad ℥xx. and a large blister applied to the affected part. ℞. Hydrargyri submuriatis, gr. v.; Jalapi pulv. gr. xii. Sit pulvis quam primum sumendus: aq. hordei ad libit. Towards the evening, the patient indicated but slight variation. Pulse slower since the bleeding: the blood resembled one mass of pus; coat of an inflammatory nature. Had one stool, accompanied with much flatus: the fæces of a grey colour, and clayey consistence. Cough rather troublesome.—Repetatur pulvis.

August the 7th.—Passed an indifferent night; during which had two small evacuations, of the same nature as yesterday. Constant inclination to vomit; tension and hardness in the hypochondrium, as before, and more painful on the slightest touch; urine thick, and brown coloured; pulse 98, and irregular; skin dry; great prostration of strength. A copious discharge from the blister, which was promoted by the application of ung. hydrarg. fort. and cantharides. Thirst excessive; is obliged to take fluid in small quantities, from dread of the stomach rejecting it. The patient's rest during the night was occasionally disturbed by a sense of suffocation, short cough, and spasmodic twitchings at the shoulder-blade and lower extremities.—℞. Massæ pil. hydrag. Pharm. London, gr. viij. mane et vespere. Aq. hordei et acid nitros ad libitum. Balneum tepidum; and a drachm of unguentum hydrargyri fortius was directed to be rubbed into the hypochondrium twice a-day.

On the approach of evening, appeared a little easier, though stomach irritable as before. No passage to-day.—Injiciatur enema domesticum, et repetatur balneum tepidum.

August the 8th.—The patient passed a tolerable night, but is necessitated to keep in one position, lying rather over to the left side, and the knees drawn towards the abdomen. Increase of pain and tension on pressure. Had a copious evacuation, of the same nature as before. Pulse quick; tongue loaded; skin hot, occasionally covered with cold perspiration. Complains of increase of pain at the navel, particularly on endeavouring to stretch himself or alter his position.—Continue the same treatment as yesterday.

Evening.—No particular alteration: has been free from nausea, and eaten a little rice for dinner.

August the 9th.—Appears much worse: great anxiety; very restless night; pain at the left hypochondrium more acute, and, on pressure, the pain at the navel and right hypochondrium insupportable; tumefaction more painful to the touch, and apparent sense of fluctuation, particularly towards the left hypochondrium. Frequent retching and inclination to vomit; increase of debility, accompanied with hectic fever; pulse quick and contracted; tongue loaded as before. Had one evacuation towards the morning.—Omitted the Mass. pil. hydrarg. et ung. hydrarg. fort. Ordered the application of an emollient cataplasm to the affected part, and tepid fomentations to the umbilical region. Injiciatur enema domesticum bis in die; and one ounce of mistura salina effervescens to be taken every two hours, with ten drops of tinct. opii.

Evening.—The patient had one scanty stool since the morning; fæces the same. Slept four hours in the day. Free from vomiting and retching; tongue a little moist; other symptoms as before.—Continuantur medic. ut mane, if no return of nausea. Ordered a wine-glass of wine and water, equally mixed, to be given at midnight and four o'clock in the morning.

August the 10th.—Passed a tolerable night; no vomiting. Had two copious evacuations of a similar nature; urine scanty, of a dark colour; fluctuation of pus more perceptible; no prominent part in the tumor. Took some toast and tea for breakfast. The patient much debilitated.—Continued the same treatment, and increased the quantity of wine to three glasses during the day, mixed with water.

Evening.—No alteration since morning. Inclined to somnolency; is occasionally disturbed with a feeling of suffocation. Continued fomentations, cataplasm, and misturæ salinæ, as before.

August the 11th.—Passed a restless night. Hectic and debility augmented; return of vomiting, emitting bilious matter; stupor, accompanied with numbness; feet are œdematous; respiration laborious; a clammy perspiration overspreads the face and neck. Pulse so low as to be hardly perceptible; eyes dull and languid, countenance pale. No evacuation since yesterday; urine still the same colour. Tumor extending down towards the umbilicus: the pus situated very deep, and is become more easily distinguished at the left hypochondrium, near the false ribs. Being fearful the matter might escape into the abdominal cavity, I ventured to embrace the moment in explaining the necessity of the abscess being opened; but, to my unfortunate patient, no entreaty or persuasion was of any avail, who thus expressed himself—"I would rather die a thousand deaths than have my liver cut to pieces, for then I am sure to die." I continued the cataplasm and fomentations. Injiciatur per anum enema anodynum mane et nocte. ℞. Infus. cuspariæ, ℥viij.; Acid muriat. ʒj. M. fiat mistura, bibat cyathum vinosum tertia quaque hora.

Rather amended as evening advanced, and less inclination to vomit; pulse feeble. Had one stool.—Continued treatment as before.

August the 12th.—No alteration. The patient appeared gradually sinking under his complaint; pain insupportable; spasmodic affections in the lower extremities; stomach more settled, pulse better, and tongue a little moist. He expressed a strong inclination for a glass of porter: half a glass allowed to be given, and the same treatment continued.

Evening.—No alteration; but says the porter has been of service to him. Allowed the same quantity as in the morning, and ordered it to be repeated at midnight.

August the 13th.—No sleep the whole night. Free from vomiting: one copious evacuation; pulse 120. The patient observes, the pain is "beyond description." The abscess is becoming more prominent towards the left side, in the direction of the spleen.—Continued the same treatment, and ordered ten drops of tinct. opii to be given with the infus. cuspariæ. I still urged every persuasive to open the abscess, by assuring him of his recovery; that, if he did not submit to the operation, death would be inevitable in four-and-twenty hours: on which the poor fellow replied, "if he survived till morning, it should be performed." I continued in the same manner, anxiously looking forward to the promised hour of relief, which he would sooner have obtained, but for obstinately deferring the operation.

Evening.—No alteration: complains of increase of pain at the clavicle, and along the spine.—Ordered twenty drops of tinct. opii to be given at midnight, in addition to the ten drops added to the infus. cuspariæ.

August the 14th.—Passed an indifferent night, at intervals dozing. Complains of great difficulty in making urine. His countenance evinced a deadly paleness. Fluctuation of pus more perceptible in the left hypochondrium, though very deep. Frequent shiverings; pulse feeble; pain about the clavicle; respiration very laborious, accompanied with singultus. Reduced to this extreme distress, and influenced by a feeling that generally characterises this class of men, and not from the faintest hope of recovery, he observed, "he should not like to depart from this world; therefore, I might cut his liver and lungs to pieces, as soon as I thought proper."

I immediately prepared the necessary dressings, laid the patient inclining to the left side, and requested the assistant surgeon to press with both hands upwards along the umbilicus, towards the left lobe of the liver, in order to confine the pus as much as possible to one point: with a scalpel I made a longitudinal incision, in the most prominent part, through the external integuments, about three fingers' breadth from the false ribs, and two inches in length, further directing the scalpel into the cyst, though very deep seated. The pus instantly discharged, exceeded two quarts, and was of a fœtid quality; when the unhappy patient joyfully expressed that he was free from pain. I then ascertained the size and direction of the abscess, by the introduction of a common bougie, measuring ten inches, which was nearly concealed: it passed in the direction of the right lobe of the liver, approaching to the navel. I observed considerable induration on both lobes, and also at the navel on pressure. A pledget of lint, spread with ceratum resinæ, was applied between the lips of the wound, over which compression was made with thick folds of linen; passing a flannel roller four or five times round the body. I ordered an anodyne and a glass of porter; the latter to be repeated in the course of two hours.

Evening.—Dressed the wound: a considerable quantity of pus, of the same nature, was discharged through the dressings and bandage. To give vent more freely to the abscess, I requested him to lie on the left side; which he did, without finding the position give any particular uneasiness. Slept five hours since morning; had one evacuation; suffers little pain. Dyspnœa and slight cough still continue; pulse 86, of tolerable strength. Dressed the wound as before. Continued the infus. cuspariæ, without tinct. opii. Habeat vini rubri uncias octo.

August 15th.—Passed such a comfortable and undisturbed night as he had not enjoyed for many months. Nausea entirely removed; ate some rice and tea for breakfast, with tolerably good appetite. Had one evacuation towards the morning, of a clay colour and greenish cast combined. Countenance much improved. A copious discharge from the abscess, of a similar nature: dressed the wound as yesterday. Continuatur medic. et habeat vini rubri libram in dies.

Evening.—No particular alteration; appetite progressing.—Ordered a glass of porter and some sage for supper. Wound as in the morning.

August the 16th.—Patient improving; sleeps tolerably well. Toward morning had one evacuation, of a more natural colour, tinged with bile. The discharge from the abscess less profuse, and of more consistence. Appetite still increasing. Pulse 80, of moderate strength; tongue moist.—Dressings, medicam.; vinum, &c. ut antea.

Evening.—No change since morning; ate some rice and broth for dinner.

August the 17th.—Great amendment. Sat up for two hours to-day. The pus from the abscess has assumed a more healthy appearance; strength advancing.—Continued the treatment as before.

August the 18th.—Sat up for five hours, and enabled to walk about the room without pain. Discharge from the abscess still improving in appearance and consistency: dressed it superficially with lint and cerat. resinæ. Bowels rather costive: ordered an ounce of ol. ricini.

August the 19th.—State much amended. Several evacuations, almost of a natural colour. Discharge from the abscess diminishing; hardness about the hypochondrium and umbilicus gradually decreasing. Omitted infus. cuspariæ.—℞. Massæ pil. hydrarg. Pharm. Lond. gr. vj. bis in dies. One drachm of the strong ointment of quicksilver rubbed in the hypochondrium at night.

August the 20th.—Sleep continues regular; strength progressing. Discharge from the abscess has assumed the appearance of healthy pus. Escharotics are occasionally applied, to keep down the fungus.—Continuatur pil. hydrarg. Two glasses of porter given daily, exclusive of wine.

From the 20th till 31st, no particular alteration.

September the 1st.—Induration at the hypochondrium and umbilicus fast diminishing; skin, natural colour; mouth rather affected from the mercury. Making rapid progress to his full strength. Omitted the ung. hydrarg. fort. and took only four grains of the mass pil. hydrargyri daily. Stools become natural; wound cicatrising: escharotics are occasionally required.

September the 10th.—Convalescent.

September the 15th.—Free from complaint; wound healed, and the patient able to move in every direction without pain. Ordered four grains of the massæ pilulæ hydrargyri to be taken three times a week, and to be continued for at least eight weeks.

September the 30th.—Hardness in the hypochondrium and umbilicus perfectly removed. The patient feels himself stronger and better than he has been for many years.

October the 4th.—Dismissed cured, and sailed for the United States of America.

While transcribing this case, I met the captain of the ship in which my late patient sailed to America, who informed me that he was enjoying perfect health on the 21st November, when he last saw him.

It was extraordinary, in an abscess of such magnitude, that the pus could not be more easily distinguished; which I attribute to such considerable induration, both over and surrounding the tumor.

The abscess was situated in the concave part of the liver; I was, therefore, necessitated to make the incision very deep, and through part of the viscus apparently sound, to enter the cyst. The professional gentlemen who assisted me were doubtful whether the tumor contained any matter. The patient's strength was so far exhausted, that I expected hourly to hear of his decease. I was likewise apprehensive that the tumor might break, and, the pus being discharged into the cavity of the abdomen, death would necessarily have ensued. Although he was at intervals relieved, this but too frequently precedes approaching dissolution, which must have been inevitable had the operation been delayed even a few hours longer. I am, therefore, of opinion, that copious bleeding is too generally resorted to after the symptoms of pus become manifest, which is ultimately injurious to the patient, who requires the utmost support. Could I have ascertained what symptoms the patient had prior to being placed under my care, I should not have had recourse to venesection. It is particularly necessary that the strength be supported by wine and cardiaca, whenever the stomach will permit. I will not hesitate to say, that many patients suffering from an abscess in the liver are lost through the timidity of the medical attendant, waiting with anxious hope for the tumor to break externally; or from extreme debility brought on by an improper use of mercury.

This is the fourth case that has occurred within my practice, where incision has saved the patient. In the three former instances, I operated in an earlier stage: consequently, the sufferings of the unfortunate subjects were less severe. Even if the result be considered uncertain, as the operation is the only resource remaining, humanity evinces it should be performed, without fearing the imputation of a fruitless attempt, unless restrained by motives unconnected with the medical attendant.

A long residence in tropical climates has afforded me ocular proofs that there exists not that difference in hepatitis in opposite climes, which medical men commonly suppose, and who hence infer that the treatment should also be dissimilar.

*Liverpool; 30th October, 1824.*

